# A novel Mixture Model Method for identification of differentially expressed genes from DNA microarray data

**DOI:** 10.1186/1471-2105-5-201

**Published:** 2004-12-16

**Authors:** Kayvan Najarian, Maryam Zaheri, Ali A Rad, Siamak Najarian, Javad Dargahi

**Affiliations:** 1 Computer Science Department, University of North Carolina Charlotte, University City Blvd, Charlotte, NC, USA; 2 Computer Engineering and IT Department, Amirkabir University of Technology, Tehran, Iran; 3 Mechanical and Industrial Engineering Department, Concordia University, CONCAVE Research Centre, CR-200, Concordia University, Quebec, Canada

## Abstract

**Background:**

The main goal in analyzing microarray data is to determine the genes that are differentially expressed across two types of tissue samples or samples obtained under two experimental conditions. Mixture model method (MMM hereafter) is a nonparametric statistical method often used for microarray processing applications, but is known to over-fit the data if the number of replicates is small. In addition, the results of the MMM may not be repeatable when dealing with a small number of replicates. In this paper, we propose a new version of MMM to ensure the repeatability of the results in different runs, and reduce the sensitivity of the results on the parameters.

**Results:**

The proposed technique is applied to the two different data sets: Leukaemia data set and a data set that examines the effects of low phosphate diet on regular and *Hyp *mice. In each study, the proposed algorithm successfully selects genes closely related to the disease state that are verified by biological information.

**Conclusion:**

The results indicate 100% repeatability in all runs, and exhibit very little sensitivity on the choice of parameters. In addition, the evaluation of the applied method on the Leukaemia data set shows 12% improvement compared to the MMM in detecting the biologically-identified 50 expressed genes by Thomas et al. The results witness to the successful performance of the proposed algorithm in quantitative pathogenesis of diseases and comparative evaluation of treatment methods.

## Background

Recently, microarray technology has provided the means for simultaneous screening and analysis of thousands of genes. Although an enormous volume of data is being produced by microarray technologies, the full potential of such technologies cannot be accessed without the ability to sift through the noisy signals to obtain useful information. The large data sets produced by microarray technology have resulted in the need for reliable, accurate, and robust methods for microarray data analysis. A major challenge is to detect genes with differentially expression profile across two experimental conditions. In many studies, the two sample sets are drawn from two types of tissues, tumours or cell lines, or at two time points during the course of a biological processes.

The computationally simple methods used for such analysis, including the methods of identifying genes with fold changes (such as the popular Log-ratio graphs) [1], are known to be unreliable due to the fact that in such methods the statistical variability of the data is not properly addressed. While various parametric methods and tests such as two-sample t-test [2] have been applied for microarray data analysis, strong parametric assumptions made in these methods as well as their strong dependency on large sample sets restrict the reliability of such techniques in microarray problems. The nonparametric statistical methods, including the Empirical Bayes (EB) method [3], the significance analysis specialized for microarray data (such as SAM [4]) and the mixture model method (MMM) [5] have been specialized and applied for microarray data analysis. It is claimed and argued that the new extensions of the MMM are among the best methods producing biologically-meaningful results [5,6]. In this paper, without ignoring the potential applicability of non-parametric methods in microarray processing applications, due to the claims made in [6], we have restricted the comparison of our methods only to the MMM based methods.

The major disadvantages of the above-mentioned methods, especially the MMM, include the lack of repeatability of the results under different runs of the algorithm, and the sensitivity of the algorithm on parameter initialization. A reliable microarray analysis method must be reproducible and applicable to different data sets under different experimental conditions. More specifically, an accurate microarray processing method is expected to pinpoint, with a relatively high degree of accuracy and robustness, genes with elevated expression levels that are related to the experimental condition in all runs. The main objective of this paper is to design and test an extension of the MMM whose results are reproducible, more biologically meaningful, and significantly less sensitive to the models' parameters.

The paper is organized as follows. In Algorithms section, a review of the MMM and its recent extensions, Mod2MMM, together with the detailed description of the proposed method are given. In Results and Discussion section, the K5M algorithm is first applied to the well-studied Leukaemia data set [7] that is often treated as a benchmark problem to compare different algorithms with each other. Once the desirable performance of the proposed algorithm is verified against the Leukaemia data set, the algorithm is applied to a new data set [[8-14] and [15]] that deals with the pathogenesis of Hypophosphatemia, which is a common X-linked metabolic bone disorder in human and mouse. Finally, the Conclusion section is in the end.

## Algorithms

### MMM & its recent extensions

We start this section with a brief review of the existing MMM based techniques. Consider *Y*_*ij *_as the expression level of gene in array *i *(*i *= 1, ..., *n*; *j *= 1, ..., *j*_1_, *j*_1 _+ 1, ..., *j*_1 _+ *j*_2_), where the first *j*_1 _and last *j*_2 _arrays are obtained under two conditions. A general statistical model for the resulting data is:

*Y*_*ij *_= *a*_*i *_+ *b*_*i*_*x*_*j *_+ *ε*_*ij *_    (1)

Where *x*_*j *_= 1 for 1 ≤ *j *≤ *j*_1 _and *x*_*j *_= 0 for *j*_1 _+ 1 ≤ *j *≤ *j*_1 _+ *j*_2_. In addition, *ε*_*ij *_is a random error with mean 0. From the above formulation, it can be seen that *a*_*i *_+ *b*_*i *_is the mean expression level of the first condition, and *a*_*i *_is the mean expression level of gene *i *in the second condition. The method requires that both *j*_1 _and *j*_2_, the number of data sets for each experiment condition, be even.

The t-test statistic type scores (2) and (3) are calculated on the pre-processed data. Here, *a*_*i *_is a random permutation of a column vector that contains *j*_1_/2 1's and *j*_1_/2 -1's and *b*_*i *_contains *j*_2_/2 1's and *j*_2_/2 -1's.





Since the data are not assumed to be normally distributed, the distribution functions *f*_0 _and *f *are estimated as in (4) and (5), respectively. The null distributions, *f*_0 _and *f*, are estimated directly in a nonparametric model for gene expression data.





Where *φ*(z; *μ*_*i*_, *V*_*i*_) symbolizes the normal density function with mean *μ*_*i*_, variance *V*_*i*_, and the mixing proportions *π*_*i *_define the linear combination of the normal basis function. We use Φ_*g*0 _to represent all unknown parameters {(*π*_*i*_, *μ*_*i*_, *V*_*i*_): *i *= 1, ..., *g*_0 _} in a *g*_0_-component mixture model. The number of normal basis functions, i.e. *g*_0 _can be estimated by the EM algorithm, which maximizes the log-likelihood function of (6) to obtain the maximum likelihood estimation of .



Within *K *iterations, the EM algorithm is expected to find the local maxima for all unknown parameters. It is recommended to run the EM algorithm several times with various random starting parameters and choose the final estimate as the one resulting the largest log-likelihood [6]. As mentioned above, using random starting points causes the result of the MMM instable and avoids reproducibility of the results. More specifically, in each run the MMM algorithm may give different number of expressed genes, which is not desirable in biological studies. This issue will be addressed in our proposed method.

After finding the optimized  for different *g*_0 _'s, the algorithm selects the sub-optimal *g*_0 _corresponding to the first local minimum of BIC or AIC [16].





where *v*_*g*0 _is the number of independent parameters in Φ_*g*0_. Then, the algorithm uses the resulting *g*_0 _as the number of normal functions to fit *f*_0_. The same procedure is performed to estimate the sub-optimal number of normal functions to estimate *f*. As mentioned above, with the fixed number of normal functions, the parameters of functions *f *and *f*_0 _are iteratively updated for a number of iterations. When the iterations are terminated, the likelihood ratio is estimated based on the final estimations of *f*_0 _and *f*:

*LR*(*Z*) = *f*_0_(*Z*) / *f*(*Z*)     (9)

A bisection method [17] with a Bonferroni adjustment is used to determine the cut-off points [18] for decision-making. This means that for a threshold value *s*, if *LR*(*Z*) <*s*, then the gene is identified to have significantly altered expression in two experiments. It is possible to determine the rejection region numerically, i.e. for any false positive rate *α*, the threshold value *s *= *s*(*α*) can be calculated from the following integral:



In literature of microarray processing, *α *= 0.01 is often used as the genome wide significant level, so the gene-specific significance level is: α* = *α*/(2*n*) Recently a new modification of the MMM algorithm, Mod2MMM hereafter, was introduced [6]. This method points out a problem in constructing the test and null statistics and indicates that the true distribution of *z *may be different from the null distribution of *Z*, which can lead to invalid inference. The modified algorithm starts with the assumption that *j*_1 _≥ 2 *j*_2 _[6], and constructs the new *z *and *Z *as you can follow in appendix1.

For the cases where *j*_1 _≥ *j*_2 _but *j*_1 _< 2 *j*_2_, *j*_1 _observations drawn under condition one are split into two equally-sized parts to calculate , *v*_*i*(1*a*) _and , *v*_*i*(1*b*) _respectively. To calculate  and *v*_*i*(2) _about *j*_1_/2 observations are drawn under condition two. While this modification can address the differences in the distributions of *f *and *f*_0_, the stability of the parameter estimation step still remains a major problem.

The main difference between the conventional MMM and its recent extensions are that the conventional MMM disregards the fact that the true distribution of *z *(the statistical variable under study) may be different from the null distribution of the statistics *Z *(as defined below). This assumption can potentially lead to invalid inference. A modified version of the MMM (Mod2MMM hereafter), introduced in [6], assumes that the denominator and the numerator of one of t-statistic-type score *_zi _*may not be independent. This method addresses the issue by constructing new *z*_*i *_and *Z*_*i *_variables as will be discussed later.

A concern over all existing MMM based methods (including Mod2MMM) that greatly affects the results is associated with the way mixed distributions are estimated. In the MMM, Expectation Maximization (EM) algorithm [19] is often used to optimize the parameters of fitted mixture distribution functions of two t-statistic-type scores related with genes expression level. Starting the EM algorithm with random values as the parameters of the normal basis functions to estimate distributions makes the results depend highly on the exact initialization, and always makes variations in the results. On the other hand, if all parameters of the normal functions in the mixture model distributions are set without iterative optimization, the set values may never result to an accurate model of the data set in hand. We propose a modified version of MMM to address this problem. Our modified MMM (K5M hereafter) combines K-mean clustering and the EM estimation to not only optimize most of the parameters with the EM iteratively but also apply K-means to optimize other sensitive parameters to ensure complete reproducibility of the algorithm. The experimental results indicate superior robustness of the proposed algorithm compared to the conventional MMM and other recently introduced extensions of the MMM [6].

### Proposed method (K5M)

In order to address the stability and reproducibility of the MMM, we propose a new modified approach for the MMM that estimates the distribution function of *z *by using mixture of normal distributions in a stable and reliable way. The following observations made in the experimental study of the MMM for gene expression analysis were the main motivations for the proposed changes to the MMM:

1 The observed variations in the parameter estimation process in some versions of the MMM can be attributed to the algorithm's attempt to iteratively update the means and variances of the normal distributions using often noisy data. In experimental studies, often the direct observation of the data reveals specific points where centers (means) can be positioned and the scattering patterns that can give reliable estimates on the variance of each cluster. However, the iterative updating of model parameters with noisy data and based on some random starting points often misses the true optimal points and even creates variations and fluctuations in parameter estimation in many runs.

2 Even when variations do not occur, two runs of the algorithm can result to significantly different estimations of *f *and *f*_0_. This in turns results to lists of differentially expressed genes in different runs. More specifically, a set of two typical runs of the algorithm on the same data set can result to two lists that are very different both in number of the genes as well as the exact genes picked up by the algorithm. In our study of the conventional MMM and Mod2MMM, two runs with the same algorithm (on the same data) resulted to lists whose size vary between 50 and 200.

3 The literature of other areas of research utilizing normal basis function for estimation including neural networks indicates that in order to have more robustness in different runs and have reproducible results, the means and variances of the basis functions must be estimated and fixed during the iteration on the coefficients [20]. This is due to the fact that updating means and variances makes the estimation process a nonlinear one that is highly sensitive and very likely to become unstable. However, when updating the values of coefficients only, the problem is reduced to a reliable linear estimation that is much more robust and stable.

4 Based on the observations mentioned above, in our proposed method, finding the distribution of *z *is regarded partially as a clustering problem, i.e. the means and variances of the normal distributions are estimated as the prototypes of a clustering step. Specifically, if *z *is distributed in a one-dimensional space, wherever there is a mass of *z*, there is a cluster with mean *μ*_*i *_and variance *V*_*i*_, which are identified by the members of that cluster.

Hence applying a clustering method is capable of estimating the means and variances of each normal distribution. The key is to use a simple clustering technique such as K-mean to estimate the mixture distributions *f*_0 _and *f *based on *K *normal distributions. While the algorithm can use K-means to find the optimal values of means and variances, the coefficients *π*_*i *_'s need to be optimized using an optimization process such as the EM.

Based on the above discussion, the proposed algorithm can be described in the following two steps:

**Step 1**: Using BIC, find the sub-optimal number of normal distributions for both *f *and *f*_0 _(as described above). These optimal numbers are then used as the number of clusters in K-means technique.

**Step 2**: Using K-means clustering technique, for both *f *and *f*_0 _find the best mean *μ*_*i *_and variance *V*_*i *_for all clusters.

**Step 3**: With the obtained values of *μ*_*i*_, *V*_*i *_and using the EM algorithm, iteratively update the values of the optimized *π*_*i *_for all clusters (both *f *and *f*_0_), i.e.



**Figure 1:**
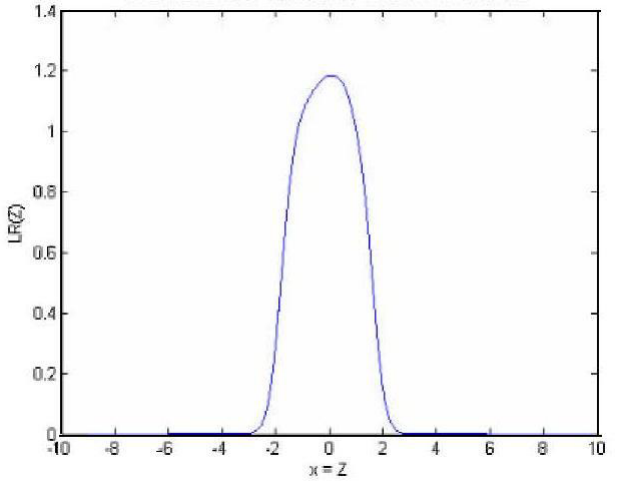
Likelihood ratio statistics as a function of Z value based on the MMM method.

**Figure 2:**
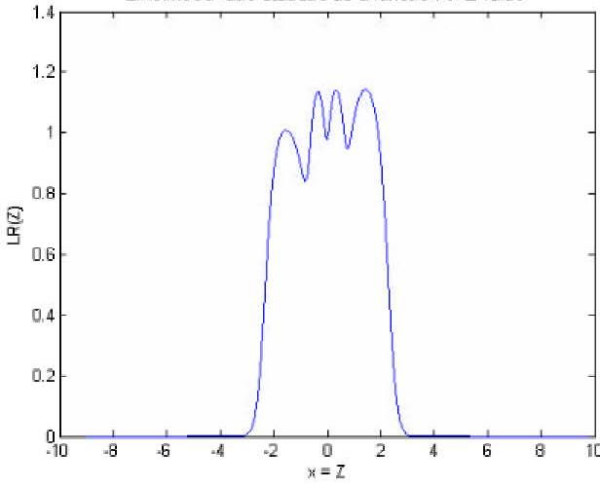
Likelihood ratio statistics as a function of *z *based on the K5M with K = 4.

**Figure 3:**
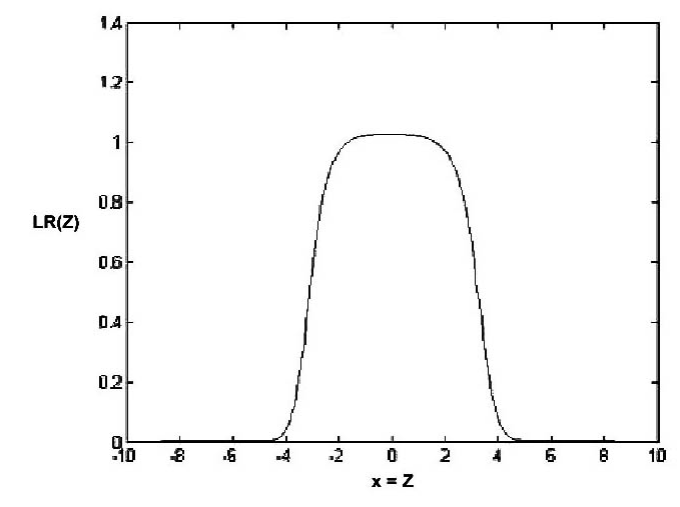
Likelihood ratio statistics as a function of *z *based on the K5M with K = 2.

A reasonable number of clusters is expected to be obtained from the first step of the algorithm, and the estimation results of the two bellow data sets in Tables 1 and 4 show that the used K (calculated based on AIC) is satisfactory. Table 3 shows the results of the MMM and K5M methods for the run with an unequal variance and four normal distributions for both *f *and *f*_0_. The MMM creates the likelihood ratio (LR) statistics plotted in Figure 1, the K5M with *K *= 4 forms the LR statistics plotted in Figure 2, and the K5M with *K *= 2 results to the LR plot of Figure 3.

**Table 1:** Comparison of the result of the K5M with the MMM and the Mod2MMM based on the Leukaemia data.

**Method**	**Total detected genes**	**ALL**	**AML**	**Total accepted genes out of 50 genes [22]**
MMM	187	21	18	39
Mod2MMM	58	14	16	30
K5M, K = 3	185	25	20	45
K5M, K = 4	58	19	8	27

**Table 3:** Estimation of fitted and by MMM (in the optimum run) and K5M.

	*f*_*0*_	*f*
MMM	= (0.1859, -0.2231, 0.0322, 0.0638)	= (-0.0387, 0.4381, 0.1600, -0.1933)
	= (0.3215, 0.3522, 0.7692, 0.337)	= (3.2288, 3.397, 2.6393, 4.6982)
	= (0.1672, 0.2353, 0.4048, 0.1925)	= (0.0687, 0.0509, 0.0263, 0.0725)
K5M	= (1.1111, 1.1264, 0.3115, -0.3329)	= (1.7867, -0.6817, -2.354, 0.3324)
	= (0.4589, 0.4640, 0.1879, 0.1807)	= (2.9432, 0.5583, 4.24, 0.5027)
	= (0.1914, 0.1963, 0.3120, 0.3001)	= (0.0583, 0.1018, 0.0294, 0.0442)

**Table 4:** The top ten most significant genes provided by K5M and MMM.

**GenBank Accession IDs**	**Gene/ Protein Description**	**Rank based on MMM**	**Rank based on K5M**
D00073	Kidney/ carrier activity	1	1
AA815845	Unknown	2	2
AF085696	ion transportation/ K+ channel, inward rectifier/renal salt flow	3	3
AW047688	Brain/Hypothalamus	4	4
M12660	Kidney/ Complement protein H gene	5	5
AI847513	Brain/ Hypothalamus	7	6
AA919924	Phosphate metabolism/inositol-1(or4)-monophospha te Activity	6	7
X69966	Dilation of the proximal renal tubules and extensive vacuolization of tubule epithelium	8	8
AF103809	Elevated kidney levels of lysosomal enzymes	9	9
AA711516	Barstead mouse myotubes MPLRB5	10	10

It is worth mentioning that due to the random initialization in K-means and the random initialization of the coefficients *π*_*i *_'s, in each run, it is expected that the number of identified differentially expressed genes fluctuate slightly. However, as indicated above, since the K- means clustering algorithm is known to a robust method, and considering the fact that in the EM estimation process, only a linear estimation is performed, it is expected that the robustness of the proposed algorithm be much more than the other version of the MMM based algorithms. This observation, as have been shown before, is supported by our experimental results. In addition, our experimental indicate that the most expressed genes are identified in all runs or the algorithm and in each run one or two new genes with less expression ratio are added to this set.

## Results and discussion

In this section, first the two applications and their corresponding data sets are described and then the results produced by the proposed method (i.e. K5M) is compared with the other MMM based methods on two data sets. The detailed description of the methods is given in MMM & its recent extensions Section.

### Leukaemia dataset

In this section, we apply the nonparametric MMM method with and without the proposed modifications to the Leukaemia data presented in [7]. The objective of this application is to identify the most important genes involved in development of different types of Leukaemia. The dataset used for this analysis includes 27 acute lymphoblastic leukaemia (ALL) samples and 11 acute myeloid leukaemia (AML) samples for 7129 genes. The main goal is to find genes with differential expression between ALL and AML cases. A second goal is to compare the result of MMM and Mod2MMM (as introduced in MMM & its recent extensions Section) with K5M and test the robustness of K5M. The genome-wide significance level is chosen *α *= 0.01 (according to Benferroni adjustment used in the MMM based methods). Each sample in the dataset is pre-processed as in [21], by subtracting its median and dividing the resulting variable by its quartile range (i.e. the difference between the first and the third quartile).

### Results of Leukaemia study

Thomas et al [22] used known biological information to identify the most important genes in Leukaemia and provided biological justifications for these identified genes. They introduced 50 genes out of the identified genes as the most expressed and related genes to the disease, including 25 most expressed genes for AML and 25 for ALL. We treat Thomas et al's list as the biology knowledge base and compare the capabilities of the computational techniques to correctly identify the genes discussed in [22] by processing the dataset.

The comparison of the result obtained by the K5M with those of the MMM and the Mod2MMM is summarized in Table 1. As can be seen in Table 1, The MMM has identified 187 differentially expressed genes [21], among which the total of 39 genes are in the list of genes obtained by Thomas et al [22]. The Mod2MMM method found 30 genes of the Thomas's list. The K5M algorithm, determines 45 genes that are identified in the Thomas's list, i.e. the proposed algorithm successfully identifies 90% of biological result. This means that K5M improved the detection of expressed genes 12% compare to the MMM and 30% compare to the Mod2MMM for the Leukaemia data, i.e. our method identified more genes from the list of the 50 truly expressed genes identified by Thomas et al [22].

As the BIC suggested the optimum number of clusters K = 4 for the MMM, the K5M is applied with K = 4 also. Running K5M with different number of clusters leads to the different but reasonably similar results. As the number of the clusters increase, the number of expressed genes decreases. Table 1 shows that the K5M with *K *= 3 identifies the total of 185 differentially expressed genes, while with *K *= 4 the total of 58 genes are identified, however; the 58 genes found with *K *= 3 are the most expressed genes among 185 genes found by *K *= 4. This result shows the consistency of the K5M method.

**Figure 5:**
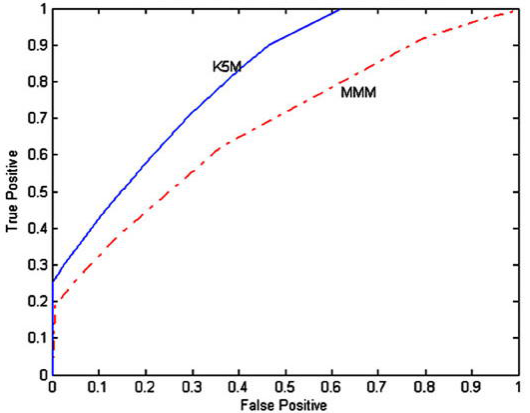
ROC curves for the MMM and K5M based on the leukaemia data set. The area under the K5M curve is more than the area under the MMM which shows the K5M method is more accurate than the MMM.

In order to further compare the performance of the MMM and K5M on the leukaemia data, The ROC curve is plotted based on False Positive rate and True Positive rate of the data set calculated as in [5]. The area under each curve is the measure of test accuracy. As can be seen in Figure 5, the area under the K5M curve is more than the area under the MMM curve, therefore the K5M is providing a more accurate classification than the MMM.

### Hypophosphatemia dataset

The following study is the main application for which the proposed method was specialized and therefore is described in more details. Hypophosphatemia is a common X-linked metabolic bone disorder in human. Hypophosphatemia results from phosphate wasting in the renal tubules. Phosphate that is normally reabsorbed from the urine is excreted. It appears that elevated levels of FGF-23 activate the excretion of phosphorous by the kidneys. Previous studies have demonstrated an impairment of the high- affinity, low capacity Na+ dependent phosphate co-transport system [23,24]. The main animal model used to study this disease is the *Hyp *mouse. *Hyp *mice have a mutation of the *Phex *gene [25,9]. The disease is characterized by low reabsorption of phosphate, bone disease, and bone abnormalities in the lower extremities. The genes active in the regulation of phosphate re-absorption in the kidney are not well understood. It is also not clear whether mutations of the *Phex *gene block renal adaptation to low phosphate diet. *Hyp *mice have a primary osteoblast defect and defects in vitamin D metabolism. Parabiosis experiments on normal and *Hyp *mice have revealed that there is an intrinsic osteoblast defect in *Hyp *mice rather than an intrinsic renal abnormality. *Hyp *kidneys transplanted into normal mice reabsorbed phosphorus at normal levels. Kidneys transplanted from normal mice into *Hyp *mice began phosphate wasting in the *Hyp *mice.

The mechanism that leads to the excessive excretion of phosphorous is unknown. On a low phosphate diet a normal mouse will activate systems to conserve phosphate by increasing re-absorption. The genes activated in the normal mouse on the low phosphate diet, and the genes with differential expression between normal and *Hyp *mice should indicate the systems involved in the phosphorus homeostasis. In an attempt to identify these genes, nutritional experiments were performed on normal and *Hyp *mice [[9,8-14] and [15]]. Normal and *Hyp *mice were placed on low phosphate diets for 3 – 5 days. Tissue samples from the kidneys of test and control mice were collected. 16 samples were analyzed using Affymetrix GeneChip mouse U74A arrays- 4 samples for each experiment state. The mRNA of 12,488 genes was analyzed. Two GeneChip microarrays were done for each diet for normal mice and three microarrays for each diet for the *Hyp *mice for a total of 10 arrays.

To investigate this, 5-week-old normal and *Hyp *were fed a control (1.0% P) or low phosphate (0.03% P) diet for five days. The four group experiments are shown in Table 2.

**Table 2:** Four experimental groups in the Hyp mice data sets. In this paper, The comparisons are done between group 1 and group2, and between group 3 and group 4.

	Diet		
	
		Control	Low Phosphate
	
Genotype	Normal	Group1	Group2
	*Hyp*	Group3	Group4

In this study, we consider the gene expression signal less than 100 as noise caused by the microarray machine, and in the pre-processing step we ignored the genes whose expression signals in both conditions are less than 100. The following two specific goals are considered in this study:

1. To identify the genes in whose mRNA expressions are altered by low phosphate diet in normal mice.

2. To determine the effect of *Hyp *mutation on this response, i.e. identifying the genes in *Hyp *condition that are differentially expressed across the normal and low phosphate diet experiments.

### Results of Hypophosphatemia study

The *Hyp *dataset includes five samples for each group. In order to make the number of data samples even, we used four samples of each group. For this data set, since j1 = j2, the Mod2MMM cannot be applied. In MMM method, five mixture models are used to estimate f_0 _and f (distributions under two experimental different conditions) with number of normal basis functions ranging from 1 to 5, i.e. The MMM algorithm was run several times and the run with maximum log-likelihood was chosen as the final model. Bayesian Information Criterion (BIC) [26] was used to determine the number of components. To find the rejection region for a given model, the bisection method is used. In this paper we assume *α *= 0.01, and therefore the gene-specific significance level used here is calculated as:

α* = 0.01/(95.44 * 2) = 5 * 10^-7^

**Figure 4:**
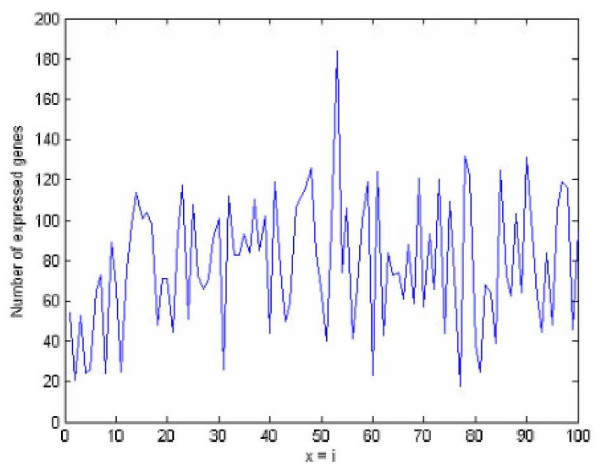
Histogram of the number of genes expressed in each run by the MMM method which shows the strong variability (x-axis shows number of runs).

Using bisection method [17], as discussed in Section 4, the value of *s *is obtained as *s *= 3 × 10_^-6^_.. Both the MMM and K5M were run 100 times. Figure 4 presents the number of genes expressed in each run of the MMM. The difference between the number of identified differentially expressed genes in two runs with the minimum and the maximum number of genes amounts to 150 genes. This clearly indicates the high degree of inconsistency and irreproducibility of the results obtained by the MMM. The number of genes expressed in each run of the K5M indicates that all genes are the same in all runs and therefore indicates 100% repeatability and robustness of the proposed method.

The ten most significant genes expressed by the low phosphate diet in the normal mouse identified by the MMM, and the ten most significant genes provided by K5M are represented in Table 4. As can be seen in Table 5, the most differentially expressed genes are same for the MMM and K5M. Out of these 10 genes, six are directly related to the kidney's functions. For this data set, the main advantage of the K5M is its consistency and robustness as discussed above. A similar procedure is conducted to accomplish the second goal of this study, i.e. identifying the role of *Hyp *condition on the most definitely expressed gene in normal and low phosphate diet microarrays. The ten most significant genes that are differentially expressed across the two experimental conditions, i.e. Normal Low Phosphate and *Hyp *Low Phosphate, are listed in table 6. As shown in the table 6, again eight genes are related directly to the kidney's function. These further witnesses to the capability of the proposed technique to discover the genes that are truly involved in the biological study.

**Table 5:** The top ten significant genes, by comparing group 3 and group 4 in table 2, provided by K5M and MMM.

**Accession IDs**	**Gene/ Protein Description**	**Rank based on MMM**	**Rank based on K5M**
AF028071	Kidney/ apical plasma membrane, Basolateral plasma membrane	3	1
D26352	Kidney/calcium ion binding	1	2
AA815845	Unknown	2	3
D00073	Kidney/ carrier activity	5	4
AB00603	Monooxygenase activity, oxidoreductase activity	9	5
U97079	GTP binding, protein binding, phosphate binding	7	6
AI315650	Detected in Kidney	6	7
X71922	Kidney/ growth factor activity, hormone activity	11	8
D43797	Kidney/carrier activity, sodium, excitatory glutamate symporter activity	Identified as a non expressed gene	9
X81059	Protein phosphate 2	Identified as a non expressed gene	10

## Conclusions

In this paper, we proposed a technique to improve the repeatability, and robustness of the mixture model method by using the K-mean clustering method in estimating the distributions. Our proposed method finds the distribution of the variables partially based on a clustering procedure and an EM optimization process. The method is applied to analyze two microarray data sets, Leukaemia data set and a data set reflecting the effect of the low phosphate diet on regular and *Hyp *mice [8] data. The experimental results indicate 100% robustness and repeatability of the results in different runs and provide 12% improvement (compared to the mixture model method) in detecting the relevant genes in both studies.

## Authors' contributions

**Maryam Zaheri, and Ali A. Rad **were in charge of writing the codes and programming aspects of the paper.

**Siamak Najarian and Javad Dargahi's **primary role was to perform a literature review on mixture model techniques, identify the aspects of the method that need to be improved, and provide suggestions to address these shortcomings.

**Kayvan Najarian's **primary roles were to design improvments to the algoritm (based on the literature review and overal modifications suggested by Siamak Najarian and Javad Dargahi), prepare and pre-process the data (for both datasets), partcipate in preperation of the *Hyp *dataset, define the *Hyp *problem interpret the results and finally write and edit the manuscript.

## Appendix 1

The Mod2MMM makes a new z and Z based on the following formula:





Where:





And:


